# Constructing Hierarchical Porous Carbons With Interconnected Micro-mesopores for Enhanced CO_2_ Adsorption

**DOI:** 10.3389/fchem.2019.00919

**Published:** 2020-01-15

**Authors:** Hainan Zhang, Zeming Wang, Xudong Luo, Jinlin Lu, Shengnan Peng, Yongfei Wang, Lu Han

**Affiliations:** ^1^School of Materials and Metallurgy, University of Science and Technology Liaoning, Anshan, China; ^2^School of Chemical and Processing Engineering, University of Leeds, Leeds, United Kingdom

**Keywords:** hierarchical porous carbons, template method, micro-mesoporous structure, activation, carbon dioxide adsorption

## Abstract

A high cost-performance carbon dioxide sorbent based on hierarchical porous carbons (HPCs) was easily prepared by carbonization of raw sugar using commercially available nano-CaCO_3_ as a double-acting template. The effects of the initial composition and carbonization temperature on the micro-mesoporous structure and adsorption performance were examined. Also, the importance of post-activation behavior in the development of micropores and synthesis route for the formation of the interconnected micro-mesoporous structure were investigated. The results revealed excellent carbon dioxide uptake reaching up 2.84 mmol/g (25^o^C, 1 bar), with micropore surface area of 786 m^2^/g, micropore volume of 0.320 cm^3^/g and mesopore volume of 0.233 cm^3^/g. We found that high carbon dioxide uptake was ascribed to the developed micropores and interconnected micro-mesoporous structure. As an expectation, the optimized HPCs offers a promising new support for the high selective capture of carbon dioxide in the future.

## Introduction

Carbon dioxide has gradually increased in the atmosphere over the past century, resulting in increasing concerns about the global warming and climate change (Kanki et al., 2016; Qi et al., 2017; Li et al., 2019). In order to reduce the carbon dioxide content in the air, people began to develop new energy, such as hydrogen energy (Qi et al., 2019), degradation of pollutants and sewage (Sun et al., 2018; Chen et al., 2019), among others, but fundamentally solve the problem of carbon dioxide pollution. However, although carbon dioxide is considered as the main greenhouse gas, it is a transformable carbon resource (Garbarino et al., 2014; Son et al., 2014; Pullar et al., 2019). In the presence of a suitable catalyst, the captured carbon dioxide molecules can be converted into synthetic natural gas, such as methane (Lavoie, 2014; Wang et al., 2016; Xia et al., 2019). Therefore, promoting the development of carbon dioxide capture and storage (CCS) has important social significance and great economic value (Han et al., 2012; Hayat et al., 2019).

Tremendous research has been devoted to the development of new technologies for CCS, especially those based on high-performance sorbents for carbon dioxide capture (Liang et al., 2004; Santis et al., 2016; Patel et al., 2017; Zhu et al., 2019). Novel solid sorbents capable of reversibly capturing carbon dioxide through dry adsorption process have many distinct advantages over wet absorption and adsorption-coupled membrane separation. These include low investment, the moderate energy cost for regeneration, and large capacity at room temperature (Yaumi et al., 2017). In the past decade, various types of solid sorbents have been attempted for CCS. Examples include zeolites (Megías-Sayago et al., 2019), modified porous silicas (Dassanayake et al., 2017), metal organic frameworks (MOFs) (Shi et al., 2019), calcium looping sorbents (Zhu et al., 2017), polymer membranes (Ahmad et al., 2016; Zhang et al., 2019), and nanoporous carbons (NCs) (Lee et al., 2014). In particular, MOFs and NCs have received much attention due to their excellent capture performance. However, MOFs are still not feasible economically and sensitive to water, resulting in failure due to structural damage.

On the other hand, NCs offer many promising applications as catalyst supports (Kim et al., 2005; Song et al., 2019), advanced electrodes (Liu et al., 2010; Xu et al., 2019), and energy-storage materials (Wu et al., 2009), among others. Various types of NCs showed promise as alternatives to carbon dioxide capture, such as modified activated carbons (Lu et al., 2008), nitrogen-doped carbon molecular sieves (Patiño et al., 2014), and hierarchical porous carbons (HPCs) (Xia et al., 2014). These NCs presented several particular features like hydrophobic surface properties, superior thermal stability, and excellent chemical resistance, as well as some potential advantages in terms of extensive sources, tunable nano-pore structure, and controllable synthesis.

In view of the physical behavior during the adsorption process, the microporous structure was found more favorable for adsorption (Fan et al., 2013). Moreover, incorporation of basic groups into carbon framework, especially nitrogen-containing species doping was found highly praised in improving carbon dioxide selective adsorption performance (Wei et al., 2013). However, the realization of both micropore-enriched structures and nitrogen-doped modification depends heavily on the raw materials and synthesis route. For instance, Lu et al. reported the direct pyrolysis of copolymers based on resorcinol, formaldehyde, and lysine using the mannich reaction to fabricated nitrogen-doped porous carbon monolith (Hao et al., 2010). They measured high adsorption capacity reaching up 3.13 mmol/g at room temperature and 1 atm. Sun et al. reported a one-pot melting-assisted strategy using resorcinol and p-phthalaldehyde as carbon precursors, melamine as a nitrogen source, and Pluronic F127 as a surfactant under self-pressurized solvent-free conditions (Zhang et al., 2015). The resulting carbon material had a hierarchical porous structure with high surface area of 748 m^2^/g, and exhibited excellent capacity of 2.73 mmol/g at 298 K and 1 bar.

From the standpoint of structural design, Qiu et al. synthesized a novel carbon sorbent with a special bimodal microporous structure (Li Y. et al., 2013). The carbonized porous aromatic framework (PAF-1) derivatives were formed by high-temperature KOH activation showed unexpectedly superior carbon dioxide capture capacity reaching up to 7.2 mmol/g at 273 K and 1 bar. However, the starting materials are seldom used and the synthesis route was complicated and cumbersome. To overcome these problems, Jaroniec et al. designed two different tailored routes to develop microporous and mesoporous carbon spheres to demonstrate that the site-occupying silica in carbon spheres works as hard templates for the large mesopores (Marszewska and Jaroniec, 2017). The resulting mesoporosity enabled faster transfer of carbon dioxide from the bulk to the micropores and effectively improved the diffusion process. The best sorbent showed carbon dioxide uptakes as high as 4.0 mmol/g (23°C, 1 bar) and 7.8 mmol/g (0°C, 1 bar). Despite the improvement in NCs, the main drawbacks related to cost and technology transfer to industrial applications remain to be solved when used for CCS. At present, the research mainly focuses on employing low-cost resources to develop efficient and economical synthesis routes for sorbents with ideal performances.

In this study, HPCs with abundant micropores were prepared using a simple and cost effective route. To optimize microporosity and mesoporosity of HPCs, several influencing factors based on the ratio of raw materials and carbonization temperature were investigated, and related synthesis mechanisms were examined. The resulting materials showed promising features toward CCS on a commercial scale.

## Experimental

### Synthesis of HPCs

The commercial nano-CaCO_3_ (ca. 40~60 nm in size from Chengdu Aike Reagent) served as a hard template and micropore producer. Raw sugar was used as the carbon precursor. HPCs were prepared by dispersing nano-CaCO_3_ in the raw sugar aqueous solution at the mass ratios of raw sugar to CaCO_3_ varying from 2:8 to 5:5. The resulting homogeneous solution was then left to evaporated and dried in a water bath at 85°C. The collected semi-dry powders were dried thoroughly and then kept at 700 or 900°C for 2 h under flowing nitrogen (99.999%). The carbonized mixture was treated with 2 M HCl solution and then washed several times with distilled water, resulting in a solid product after drying overnight at 100°C. For comparison, a tentative sample was also obtained via post-activation method for HPCs at 700^o^C for 1 h under flowing carbon dioxide (50 cm^3^/min). The as-made carbon materials were labeled as HPCs^*^-*x*-*y*, where the superscript * represents the activated sample, and lowercase *x* and *y* are the share of raw sugar in dried raw materials and carbonization temperature, respectively.

### Characterization and Evaluation of HPCs

Thermogravimetric (TG) analysis was carried out using a thermal analysis system (STA 449/F3, Netzsch) with heating rate of 10°C /min under nitrogen flow. The carbon yield of HPCs samples was calculated from the weight of resultant carbons divided by the weight of dried raw materials. X-ray powder diffraction (XRD) pattern was measured on a diffractometer (Bruker D8 ADVANCE) with a scanning rate of 2°/min, using Cu Kα radiation. The microstructure of samples were observed by field emission scanning electron microscopy (FESEM, Carl Zeiss, Supra 40). The structures and morphologies of the activated sample were characterized by transmission electron microscopy (TEM) (JEM-2100F JEOL, 200 kV). The microporosity and mesoporosity properties of samples were analyzed by the nitrogen adsorption-desorption isotherms at −196°C (Micrometrics, ASAP 3020). Specific surface areas of samples were determined using the Brunauer-Emmett-Teller (BET) equation. The total pore volume was calculated from the nitrogen adsorption amount at the relative pressure of 0.995. The mesoporous volumes of samples were determined using the Barrett-Joyner-Halanda (BJH) method. The micropore area and volume were estimated by the t-plot method (Brunauer et al., 1938). The pore size distributions were derived from the adsorption branch of nitrogen isotherms using the BJH method (Sing, 1985). The carbon dioxide adsorption performance of the sample was measured at 25 and 0°C using a carbon dioxide adsorption instrument (JWGB, JW-BK112). Prior to adsorption measurement, all samples were degassed at 200°C for 12 h.

## Results and Discussion

### TG Analysis

**Figure 1:**
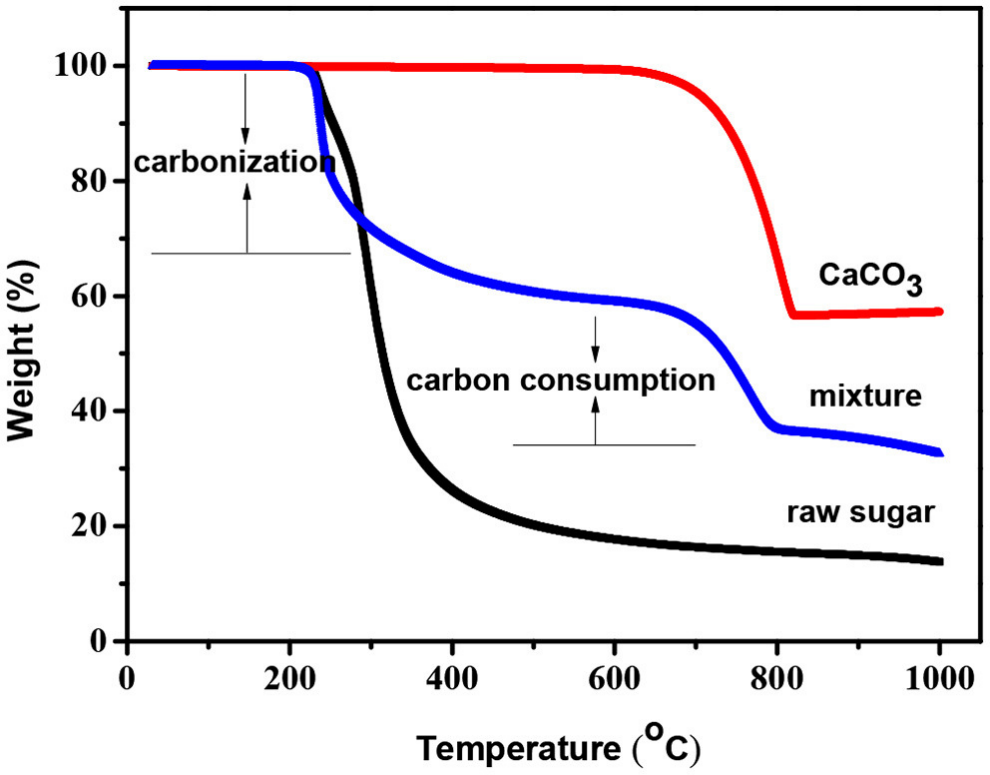
TG curves of raw sugar, nano-CaCO_3_, and raw sugar-CaCO_3_ mixture (ratio of raw sugar:CaCO_3_ = 5:5) under nitrogen flow.

The carbonization process of raw sugar-CaCO_3_ mixtures was studied by TG analysis in the temperature range from 25 to 1,000^o^C under nitrogen flow, and the results are shown in Figure 1. The decomposition temperature of nano-CaCO_3_ was estimated to about 660°C, and carbonization of pure raw sugar occurred at around 200°C with a carbon yield of ca. 19% at 900°C. Compared to pure raw sugar, the decomposition of the mixture was delayed from 300 to 660°C. This was attributed to incorporated nano-CaCO_3_ in the matrix. Above 660°C, the weight loss dropped rapidly, indicating that carbon consumption process occurred above 660°C. Since the decomposition of CaCO_3_ into CaO and carbon dioxide above this temperature, it can be concluded that carbon dioxide gas reacted with carbon walls, leading to its oxidation into CO and removal. This etching process was known as “inner-activation” effect, which was first detected in the carbonization process of PF (phenol formaldehyde) resin and CaCO_3_ composite (Zhao et al., 2010). To investigate the porous texture of the prepared carbons, two series were prepared by changing the carbonization temperature: HPCs-*x*-700 series (*x* = 2, 3, 4, and 5) carbonized at 700°C as the initial stage of etching reaction and HPCs-*x*-900 series (*x* = 2, 3, 4, and 5) carbonized at 900°C as the mature stage of etching reaction.

**Figure 2:**
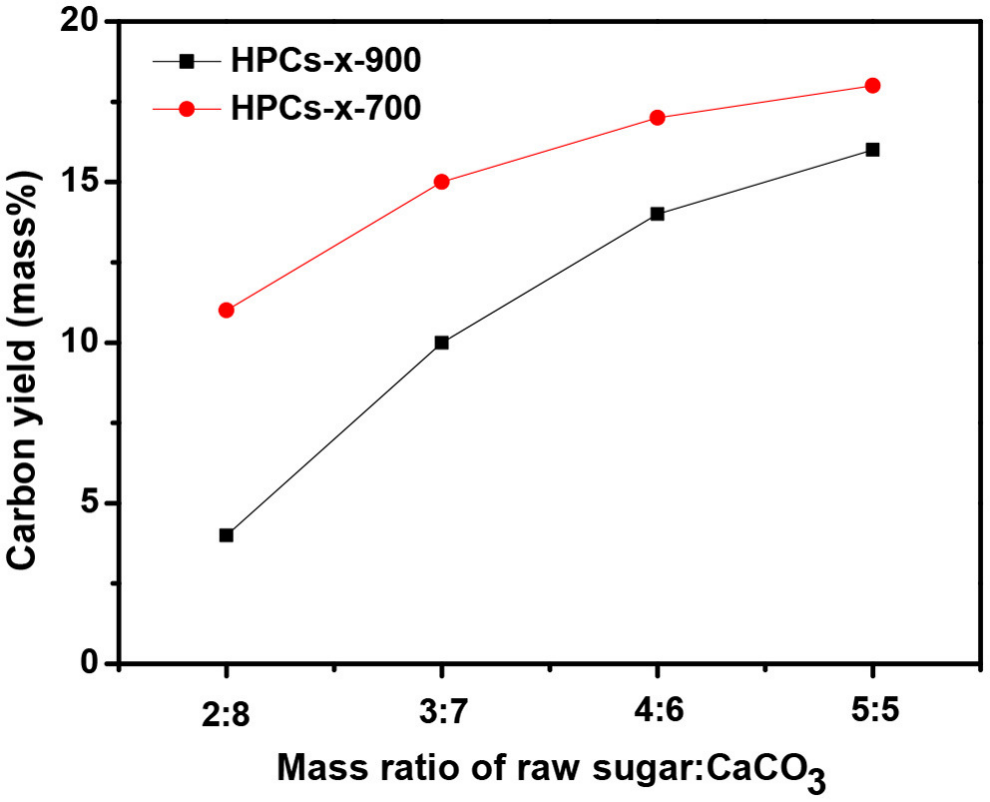
Carbon yield of HPCs-*x*-900 and HPCs-*x*-700 samples as a function of the mass ratio of raw sugar:CaCO_3_.

The carbon yield of both HPCs-*x*-700 and HPCs-*x*-900 series (*x* = 2, 3, 4, and 5) as a function of the mass ratio of raw sugar:CaCO_3_ is shown in Figure 2. Now that carbon dioxide occurred above 700°C, adequate carbon dioxide might consume more carbon according to the reaction: CO_2_+C → 2CO (Zhao et al., 2010). Figure 2 confirmed this assumption, showing that both series with a total of eight samples consumed carbon according to the above reaction. At the same initial composition, more carbon dioxide were produced as carbonization temperature rose. Thus, the carbon consumption of HPCs-*x*-900 series was higher than that of HPCs-*x*-700 series. At the same carbonization temperature, the carbon consumption gradually decreased as CaCO_3_ ratio in the mixture reduced. Thus, an increase in carbon yield was linked to the decrease of carbon dioxide emissions. The effect of carbon dioxide post-activation on porous texture of various carbons had been studied thoroughly (Liou, 2010; Li Y. et al., 2013; Wickramaratne and Jaroniec, 2013a), and the effect of inner-activation on microstructure of HPCs materials needs further investigation as well.

### XRD Patterns

**Figure 3:**
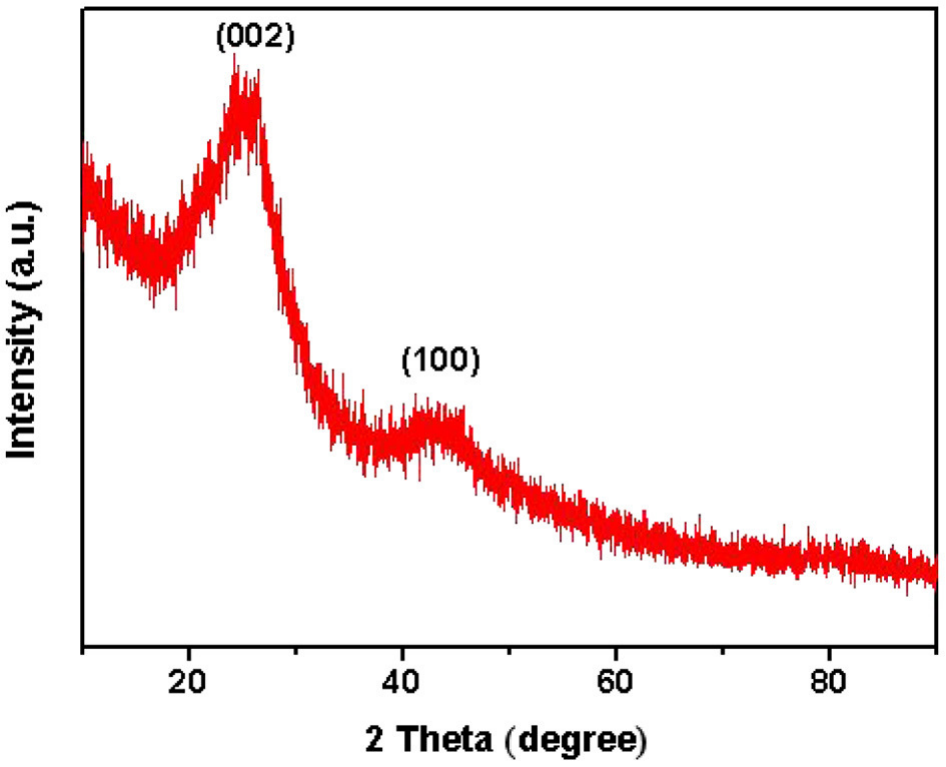
XRD pattern of HPCs-5-900.

Typical XRD pattern was exhibited in Figure 3 to verify the graphitic characteristics of carbon. A sharp diffraction peak located at 2θ ≈ 23° and a broad diffraction peak located at 2θ ≈ 44° were clearly identified, indexed to the (002) reflection of pure graphitic lattice and the (100) reflection of the graphitic carbon, respectively (Xu et al., 2011). This structure might be beneficial to enhance catalytic effect for carbon dioxide conversion when the carbon combines with active materials (Wang et al., 2016).

### FESEM Images

**Figure 4:**
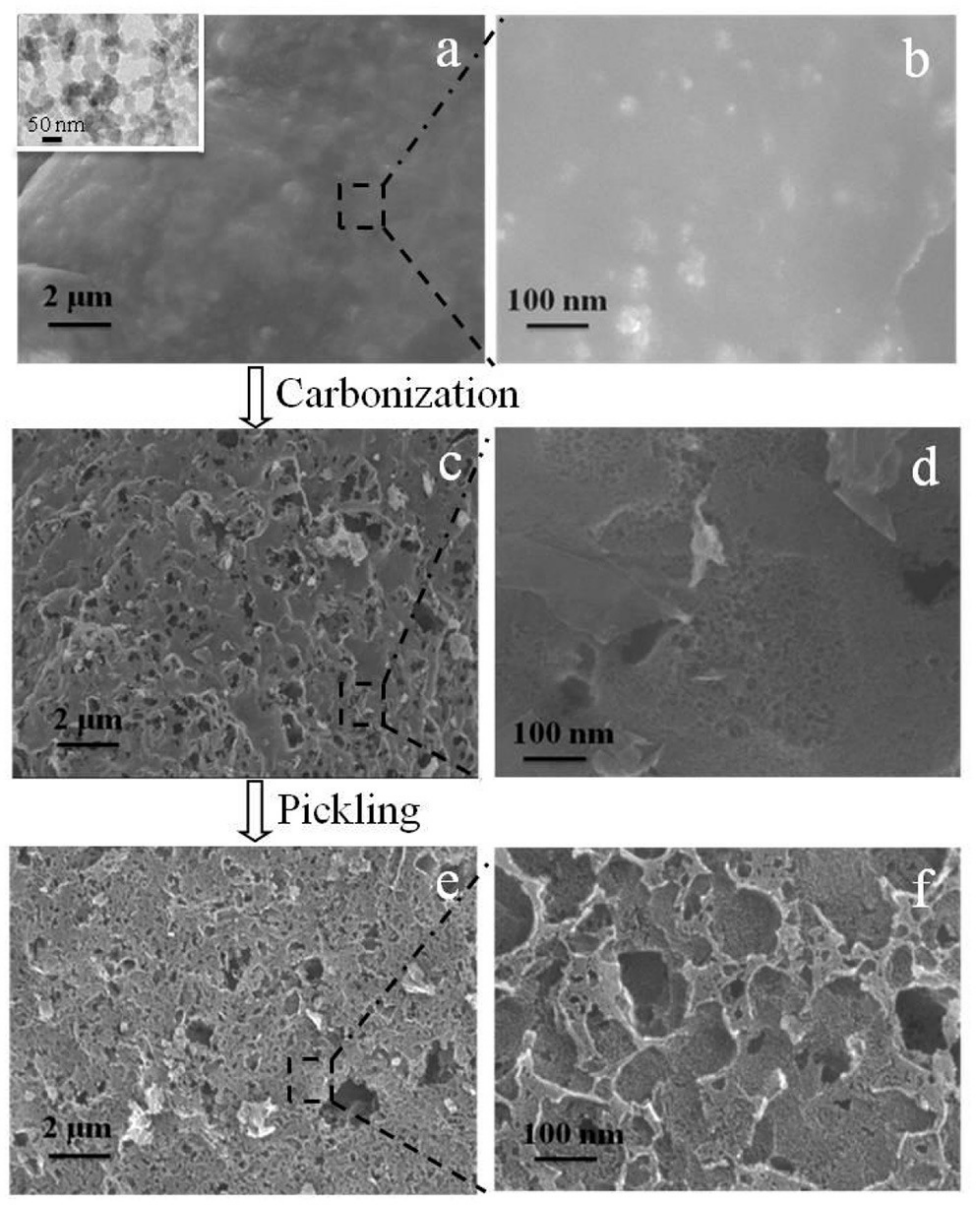
FESEM images of **(a)** and **(b)** raw sugar-CaCO_3_ mixture matrix (inset of **(a)** is the TEM image of nano-CaCO_3_ particles), **(c,d)** carbonized matrix embedded with templates, and **(e,f)** HPCs.

The morphological evolution from raw sugar-CaCO_3_ physical mixture into HPCs was depicted in Figure 4, using a series of FESEM images (a~f). In Figures 4a,b, the pristine raw sugar displayed a dense structure filled with apparent inclusions, corresponding to the nano-CaCO_3_ particles with their aggregates shown in the inset of Figure 4a. After high temperature carbonization above 700°C, the raw sugar was converted into graphitic carbon as demonstrated by TG and XRD, and the nano-CaCO_3_ decomposed into nano-CaO and carbon dioxide as confirmed by TG. As shown in Figures 4c,d, the as-received product depicted highly porous structure rich with fine pores, retaining no memory of pristine raw sugar-CaCO_3_ structure. After removal of CaO fillers, largely interconnected mesopores of 20~100 nm in diameter were formed in the carbon matrix, which were different from the morphology of matrix embedded with nano-CaO particles.

### TEM Images

**Figure 5:**
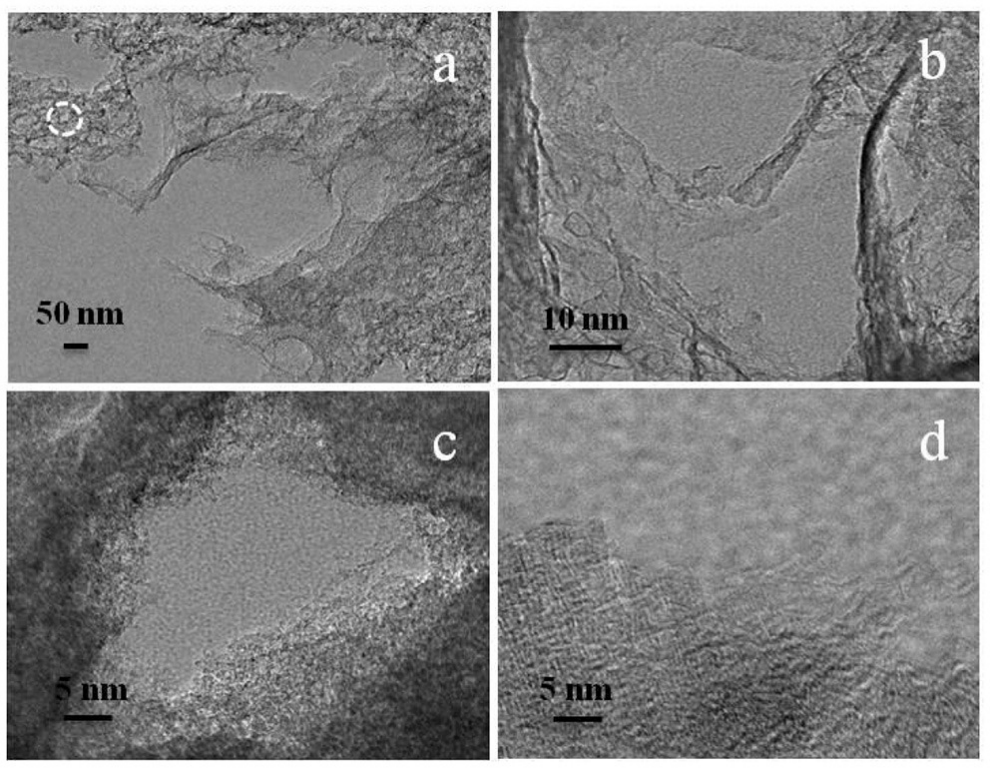
TEM images of HPCs*-5-900 showing: **(a)** edges of carbon, **(b)** randomly carbon walls, **(c)** site-occupying region, and **(d)** micropore-enriched edges after carbon dioxide gas activation.

To further investigate the microstructure of micropore-enriched HPCs after activation with carbon dioxide gas, a typical HPCs^*^-5-900 sample was characterized by TEM. As shown in Figures 5a,b, the edges at high magnification displayed well-developed three-dimensional system of the interconnected pores built by random carbon walls <10 nm in thickness to produce loose nanostructures. In Figures 5c,d, the magnified TEM image of the circular region by white circles in Figure 5a. The pore region was detected by the reaction of dilute hydrochloric acid solution with site-occupying template. The inwall of carbon was rough and curly, implying the structural disintegration of carbon and existence of fine micropores. The external carbon structure illustrated a dendritic arrangement with abundant micropores, suggesting the successful activation resulting from extrinsic carbon dioxide against carbon.

### Nitrogen Adsorption/Desorption Isotherms

**Figure 6:**
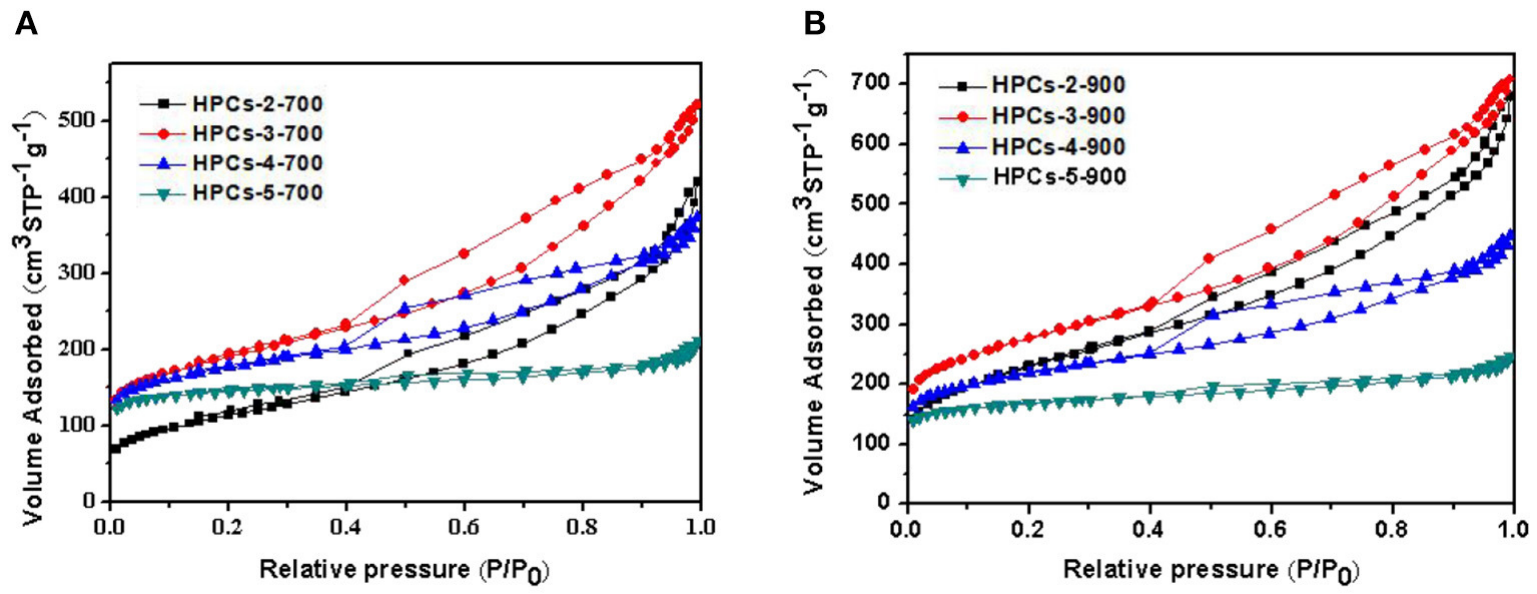
Nitrogen adsorption/desorption isotherms of HPCs samples carbonized under nitrogen at **(A)** 700°C and **(B)** 900°C for 2 h.

In Figures 6A,B, the nitrogen adsorption/desorption isotherms of HPCs-*x*-700 and HPCs-*x*-900 series (*x* = 2, 3, 4, and 5), respectively. The nitrogen adsorption/desorption isotherms of two series were typical Langmuir IV curves with distinct hysteresis loops, indicating the presence of mesopores created by the removal of site-occupying CaCO_3_ or CaO. The significant increase of adsorption volume at low-pressure range (P/P_0_ < 0.1) indicated the existence of abundant micropores. Three imaginable and reasonable sources caused the micropores to form: the voids derived from high-temperature pyrolysis of raw sugar, carbon dioxide escape routes during CaCO_3_ decomposition, and fine etching pores in carbon wall. Although there is no adsorption hysteresis in the macropore range (> 300 nm), the existence of micron level voids in the carbon matrix was clearly confirmed in the preceding FESEM images. Thus, it could be suggested that the formation of voids was mainly attributed to leaching of the aggregates of template particles (Li T. et al., 2013).

Table 1 lists the calculated adsorption parameters for both series carbonized at 700 and 900°C, respectively. All HPCs samples showed high surface areas ranging from 408 to 986 m^2^/g and large total pore volumes varying from 0.327 to 1.095 cm^3^/g. For HPCs-*x*-700 or HPCs-*x*-900 series (*x* = 2, 3, 4, and 5), as *x* increased, V_meso_ decreased gradually but V_micro_, V_micro_/V_total_, S_BET−micro_, and S_BET−micro_/S_BET_ rose gradually. This indicated a regular transition from mesoporosity to microporosity when mass ratio of raw sugar/CaCO_3_ increased. The former was attributed to the decrease in the amount of site-occupying CaCO_3_. For each HPCs series, both micropore volume and surface area increased as raw sugar ratio rose. As suggested by Figure 2, carbon yield rose as raw sugar ratio increased, in accordance with the changed trend of micropore volume and surface area. The inner-activation effect of carbon dioxide on the carbon matrix had also been confirmed by the previous discussion. The etching effect was greatly determined by respective content and dependency state of carbon dioxide and carbon, according to the reaction: CO_2_+C → 2CO. Thus, it can be concluded that the development of micropores did not only depend on carbon dioxide discharge derived from inner-activation but also closely associated with the essential amount of carbon matrix. Note that data presented here were slightly different from those reported in previous research (Zhao et al., 2010), and could be attributed to differences in the carbon source. In fact, the carbon source used in previous studies was phenolic resin. The effect of other carbon sources, such as resorcinol-formaldehyde mixture, sucrose, phenolic resin and polyacrylonitrile on porous texture will be reported in future publications.

**Table 1:** Textural properties of all related HPCs samples.

**Sample**	**S_BET_** (${\text{S}}_{\text{BET}-\text{mi}}^{\text{a}}$) **(m^**2**^/g)**	**S_**BET-mi**_/S_**BET**_**	**V_**t**_ (${\text{V}}_{\text{mi}}^{\text{b}}$) (cm^**3**^/g)**	${\text{V}}_{\text{me}}^{\text{c}}$	**V_**mi**_ /V_**t**_**
HPCs-2-700	408 (59)	0.145	0.650 (0.024)	0.626	0.037
HPCs-3-700	684 (252)	0.368	0.714 (0.107)	0.607	0.149
HPCs-4-700	646 (375)	0.580	0.578 (0.156)	0.422	0.270
HPCs-5-700	562 (491)	0.874	0.327 (0.195)	0.132	0.596
HPCs-2-900	822 (231)	0.281	1.052 (0.100)	0.952	0.095
HPCs-3-900	986 (383)	0.388	1.095 (0.165)	0.930	0.151
HPCs-4-900	793 (457)	0.576	0.693 (0.193)	0.500	0.278
HPCs-5-900	638 (532)	0.834	0.382 (0.215)	0.167	0.563
HPCs*-5-900	936 (786)	0.840	0.553 (0.320)	0.233	0.579

*^a^The micropore surface area was estimated using the t-plot method*.

*^b^The micropore volume was estimated using the t-plot method*.

*^c^The mesopore volume was estimated by BJH method*.

Moreover, the HPCs-*x*-900 series exhibited higher S_BET_, S_BET−micro_, and V_micro_ than those samples with the same raw materials ratio carbonized at 700°C, but the carbon yield of HPCs-*x*-700 series was higher than that of the carbonized samples at 900°C. This demonstrated that carbonization at 900°C could ensure the complete decomposition of CaCO_3_, and samples were thoroughly activated. In addition, the gap in amount of micropores between every two samples with the same raw materials ratio but carbonized at 900 and 700°C, respectively, became narrow due to the weakening of carbon dioxide inner-activation effect with decrease in amount of CaCO_3_. Specifically, the difference in micropore volume between HPCs-2-900 and HPCs-2-700 was estimated to 0.076 cm^3^/g. However, these values decreased to 0.058, 0.037 and 0.020 cm^3^/g, respectively, as the value of *x* rose from 3 to 5. Also, S_BET−mi_/S_BET_ and V_mi_ /V_t_ increased to 0.834 and 0.563, respectively, as the value of *x* rose to 5. The microporosity structure was more prominent, especially for HPCs-5-700 and HPCs-5-900 samples.

**Figure 7:**
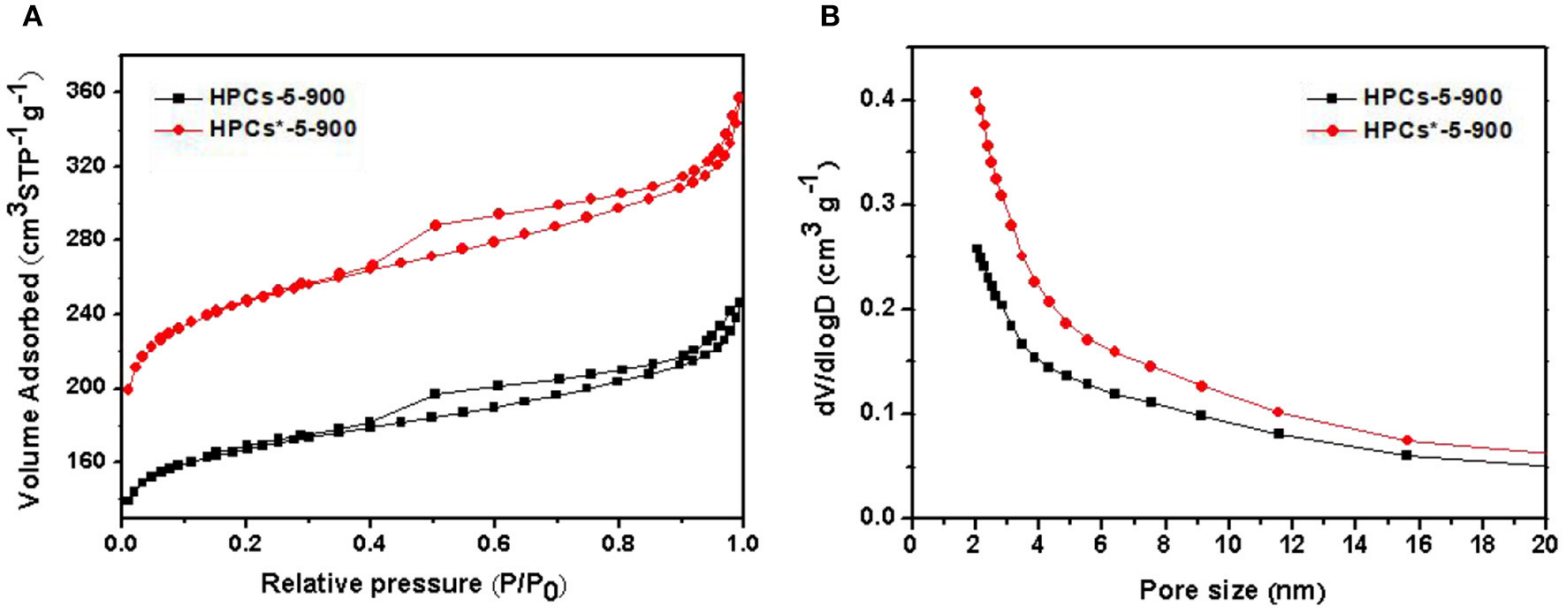
**(A)** Nitrogen adsorption/desorption isotherms of HPCs-5-900 and HPCs*-5-900. **(B)** The corresponding pore size distributions curves calculated by BJH method.

To develop large volumes of small micropores for high carbon dioxide uptakes, the carbon dioxide activation was performed on HPCs-5-900 at 700^o^C for 1 h under flowing carbon dioxide. Figure 7A shows the nitrogen adsorption/desorption isotherms of HPCs-5-900 and HPCs^*^-5-900 samples. HPCs^*^-5-900 illustrated Langmuir IV type curves with distinct hysteresis loop, indicating the presence of constricted mesopores, similar to that of HPCs-5-900 sample. As expected, the nitrogen adsorption amount of HPCs^*^-5-900 in the low-pressure range (<0.1) was significantly higher than that of HPCs-5-900 because the former was activated by carbon dioxide and its micropore volume is enlarged by almost 50%. The pore size distributions of HPCs-5-900 and HPCs^*^-5-900 samples obtained by BJH method are inserted in Figure 7B. It clearly suggested that the amount of micropores in the activated sample was superior to that of the unactivated sample, demonstrating the benefits of carbon dioxide post-activation. On the other hand, a slight increase in the mesopore volume was carefully observed from the hysteresis loop and the data listed in Table 1. The development of mesopores was also caused by carbon dioxide activation that widened all pores existed in HPCs samples or produced new pores in the carbon matrix. In other words, a part of micropores connected to become mesopores or newborn mesopores directly germinated in the carbon matrix when carbon dioxide etching reaction occurred (Li T. et al., 2013; Marszewska and Jaroniec, 2017).

### Carbon Dioxide Adsorption Isotherms

**Figure 8:**
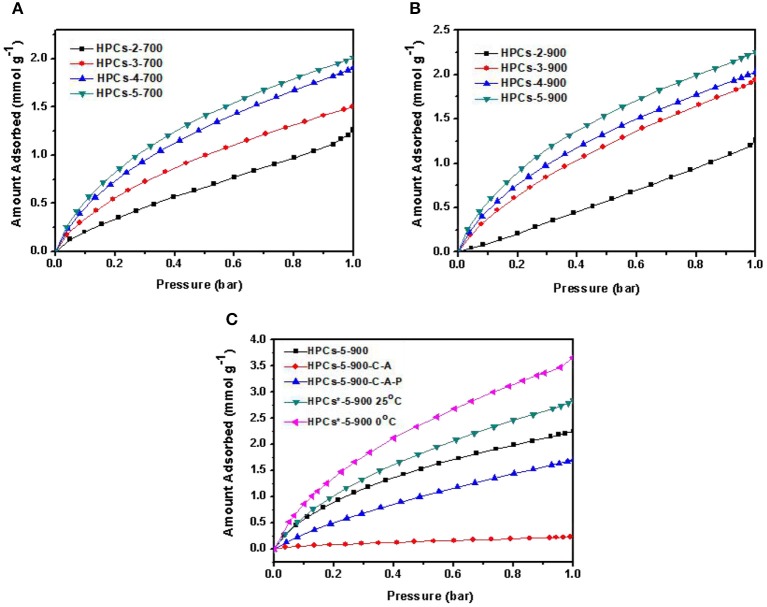
Carbon dioxide adsorption isotherms measured at 25°C for HPCs series carbonized at **(A)** 700°C and **(B)** 900°C. **(C)** Carbon dioxide adsorption isotherms measured for a series of HPCs-5-900 samples obtained by different routes.

The carbon dioxide adsorption isotherms of HPCs-*x*-700 and HPCs-*x*-900 series at 25°C are shown in Figures 8A,B, respectively. HPCs-5-900 showed the best performance toward carbon dioxide adsorption and the maximum value of adsorption capacity was estimated to 2.25 mmol/g at 25^o^C and 1 bar. Although HPCs-3-900 depicted the highest total pore volume of 1.095 cm^3^/g and mesopore volume of 0.930 cm^3^/g (Table 1), HPCs-5-900 possessed a superior micropore volume of 0.215 cm^3^/g, occupying ~56% of total pore volume. Hence, it can be concluded that the highest carbon dioxide adsorption performance of HPCs-5-900 necessarily originated from its huge amount of micropore volume. Despite using fillers, such as SiO_2_ (Feng et al., 2014; Marszewska and Jaroniec, 2017), MgO (Meng and Park, 2012) and porous concrete (Günther et al., 2012) in the preparation of porous carbon materials, nano-CaCO_3_ particles showed preferable advantages over other oxide fillers for hierarchical porous structure because they served simultaneously as mesopore templates and micropore producers (Zhao et al., 2010; Liu et al., 2013). The decomposition products of nano-CaCO_3_ particles results in nano-sized CaO particles and carbon dioxide gas, responsible for the formation of mesopores and micropores in carbon matrix, respectively. Thus, a series of HPCs with varying degrees of microporosity and mesoporosity can easily be obtained by adjusting the ratio of raw sugar/CaCO_3_ and carbonization temperature. From an economic standpoint, the mass ratio of raw sugar/CaCO_3_ as 1:1 is favorable. Besides, development of microporosity for carbon dioxide adsorption by setting the carbonization temperature at 900°C is desirable.

The chemical adsorption of carbon dioxide on HPCs materials is almost impossible due to the absence of basic groups like nitrogen-containing species in carbon. Since HPCs^*^-5-900 possessed a typical hierarchical porous structure with ultra-high micropores and moderate mesopores, and also exhibited a superior carbon dioxide uptake, it clearly demonstrated that the physical uptake was closely related to this micro-mesoporous structure. The importance of mesopores in carbon dioxide capture was recently reported (Wickramaratne and Jaroniec, 2013a,b; Marszewska and Jaroniec, 2017). In the case of HPCs materials, the synthetic route was adjusted in this study to deduce the necessity of mesopores in micropore-enriched carbon. As shown in Figure 8C, compared to HPCs-5-900, the sample obtained by preliminary carbonization, subsequent activation and final pickling (labeled as HPCs-5-900-C-A-P) exhibited a smaller carbon dioxide uptake of 1.69 mmol/g (25 ^o^C, 1 bar). Specifically, the carbon dioxide uptake of HPCs-5-900-C-A (carbonized and activated without the pickling process) just reached to 0.17 mmol/g at 25 ^o^C and 1 bar. The CO_2_ adsorption properties of the prepared all related HPCs samples are shown in Table 2.

**Table 2:** CO_2_ adsorption properties of all related HPCs samples.

**Sample**	**HPCs-2-700**	**HPCs-3-700**	**HPCs-4-700**	**HPCs-5-700**	**HPCs-2-900**	**HPCs-3-900**	**HPCs-4-900**	**HPCs-5-900**	HPCs*-5- 900
**N*^a^CO*_2_** **(mmol/g)**	1.26	1.5	1.91	2.02	1.26	1.94	2.03	2.25	2.84^b^/3.66^c^

a *The carbon dioxide uptake at 25°C and 1 bar*.

b,c *The carbon dioxide adsorption performance was measured at 25 and 0°C, respectively*.

The essential difference from HPCs-5-900, which was just the process order, would definitively affect the carbon dioxide adsorption performance. The presence of site-occupying CaO particles in carbon matrix caused two negative effects on carbon dioxide diffusion. One was related to the post-activation effect and the other was linked to the development of interconnected micro-mesoporous structure. The presence of template particles blocked activation gas diffusion from the bulk phase to the interior, resulting in delayed pore-creating reaction. For HPCs-5-900-C-A, the almost negligible carbon dioxide uptake resulted from both effects. In the case of HPCs-5-900-C-A-P, the removal of these particles during the last step resulted in interconnected micro-mesoporous structure and improved carbon dioxide diffusion from the bulk phase to micropores. Therefore, the carbon dioxide uptake increased dramatically compared to HPCs-5-900-C-A, but still lower than HPCs-5-900. Thus, the removal of site-occupying templates before carbon dioxide activation was beneficial for activation gas transfer in unobstructed path and played a significant role in improving the microporous textures. The obtained hierarchical porous structure was very important in improving the carbon dioxide uptake. In addition, as shown in Table 3, we compared the texture properties of samples in this work with the carbon samples of different structures reported in the previous literature.

**Table 3:** The comparison of textural properties of samples in this work with the literature values.

**Carbon structure**	**T (^**°**^C)**	**S_**BET**_ (m^**2**^/g)**	**V_**t**_ (cm^**3**^/g)**	**V_**mi**_ (cm^**3**^/g)**	**N_***c*_*o*__2_**_ (mmol/g) (Condition)**	**References**
Hierarchical porous carbons	900°C	936	0.553	0.32	2.84 (25°C 1 bar)	This work
Hierarchical macroporous nitrogen-doped carbons	600°C	682.8	−	−	2.69 (25°C 1 bar)	Li et al., 2016
Hierarchical porous graphene-based carbons	850°C	459	1.17	0.11	1.76 (0°C 1 bar)	Xia et al., 2014
Ordered mesoporous carbon	850°C	1400	1.02	0.42	2.64 (25°C 1 bar)	Mahurin et al., 2014
Nitrogen-doped microporous carbons	800°C	2567	1.49	1.25	2.78 (25°C 1 bar)	Fan et al., 2013
Nitrogen-doped mesoporous carbons	600°C	537	0.47	0.17	2.8 (25°C 1 bar)	Wei et al., 2013
Mesoporous carbon	900°C	3325	2.25	0.98	2.4 (25°C 1 bar)	Huang et al., 2016
Mesoporous carbon	800°C	974	1.93	0.1	2.41 (25°C 1 bar)	Lee et al., 2014
Mesoporous carbon	800°C	748	0.49	0.23	2.73 (25°C 1 bar)	Zhang et al., 2015

## Conclusions

In this study, a series of high-quality and cost-effective carbon dioxide sorbents based on HPCs are successfully synthesized by carbonizing raw sugar-CaCO_3_ composite precursors. Both starting materials are mass-produced and low-cost and the whole fabrication process only involved three steps. The carbonization and inner-activation reaction occurred synchronously, and their combination effectively induced regional micropores. The removal of site-occupying templates ensured the formation of the interconnected micro-mesoporous structure built by ultrathin carbon walls. The well-developed micropore-enriched structure was further improved through the final post-activation process. The combination of nano-CaCO_3_ incorporation and carbon dioxide post-activation were found to have significant beneficial effects on the structural controllability, especially the microporosity and mesoporosity. The best structural property with t-plot micropore surface area of 786 m^2^/g and micropore volume of 0.320 cm^3^/g were translated into excellent carbon dioxide uptake of 2.84 mmol/g at 25°C and 3.66 mmol/g at 0°C. Overall, the proposed synthesis methodology of HPCs looks promising for future applications in the field of carbon dioxide catalytic conversion.

## Data Availability Statement

All datasets generated for this study are included in the manuscript.

## Author Contributions

LH was the most important contributor of manuscript, and other authors have made a lot of contributions in experiments and writing of manuscript.

### Conflict of Interest

The authors declare that the research was conducted in the absence of any commercial or financial relationships that could be construed as a potential conflict of interest.
